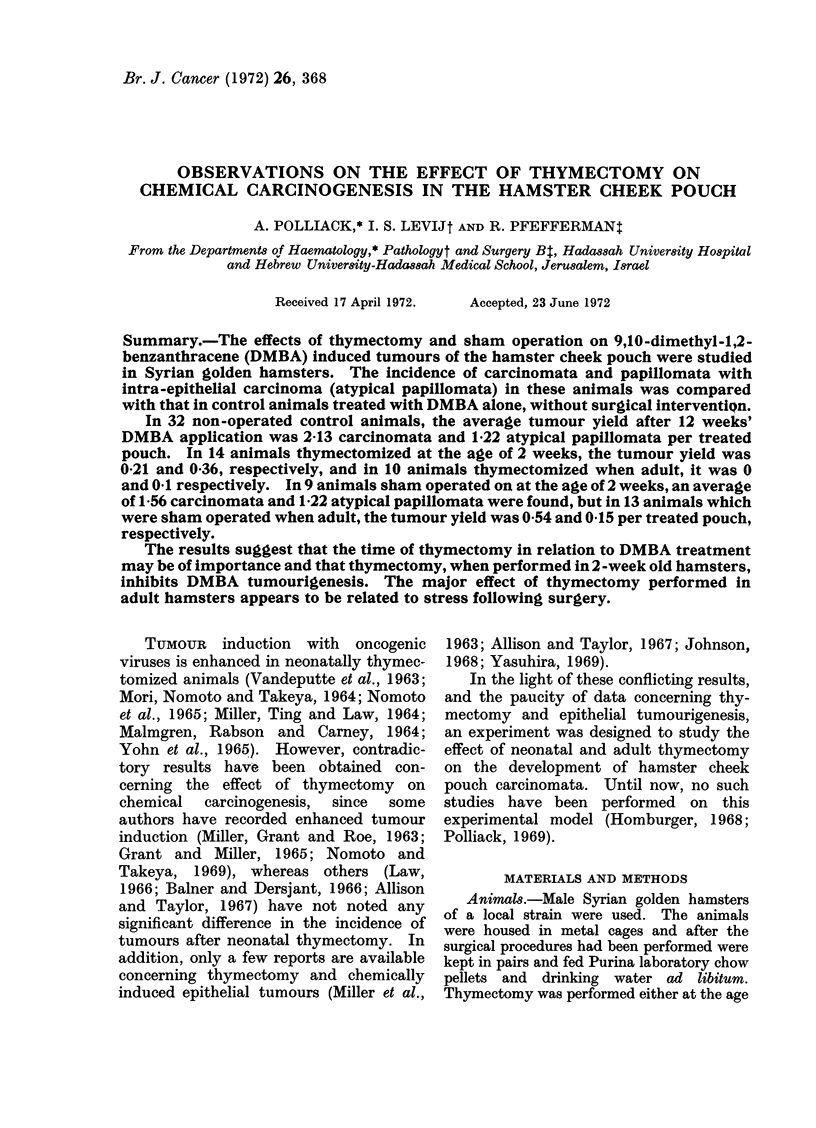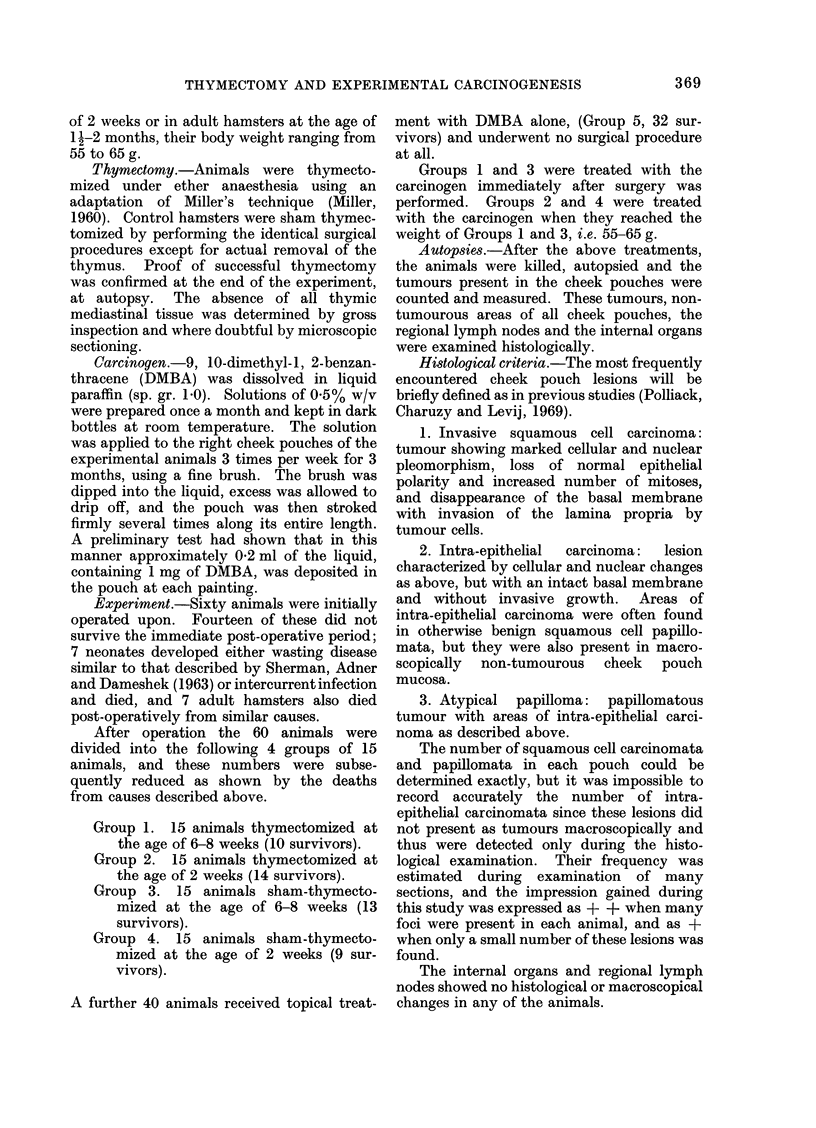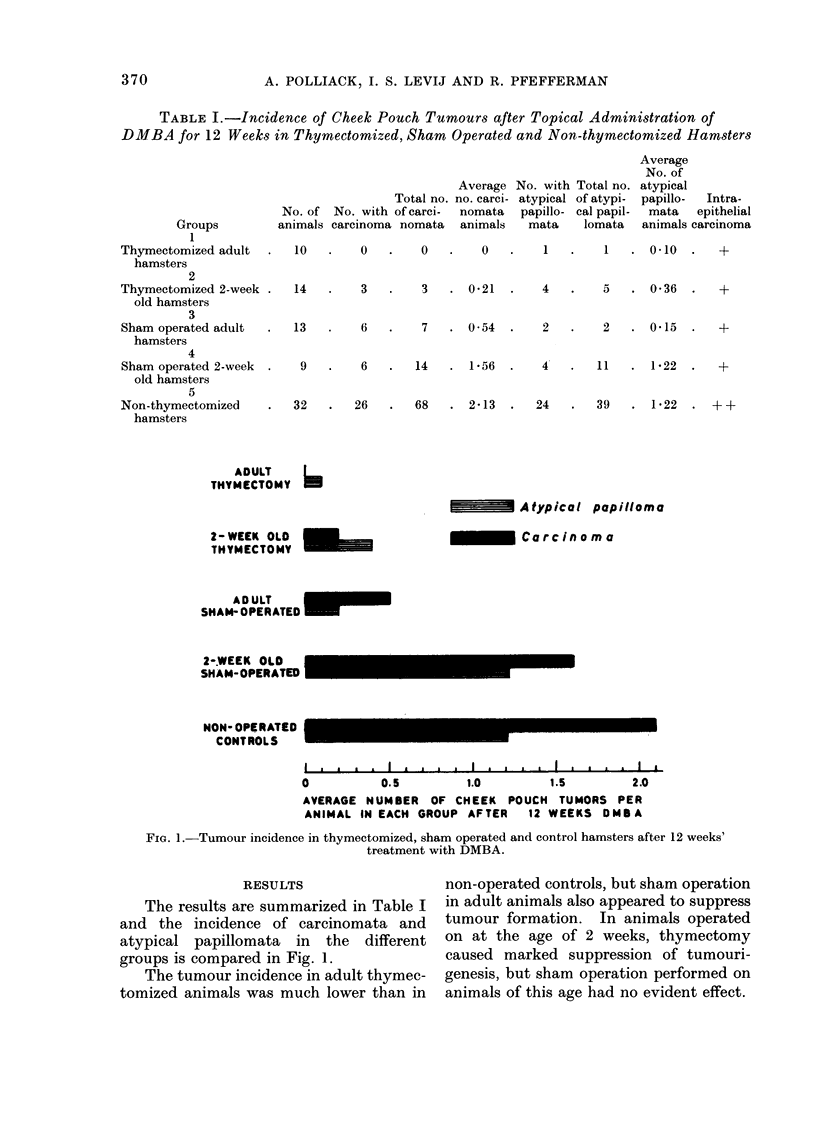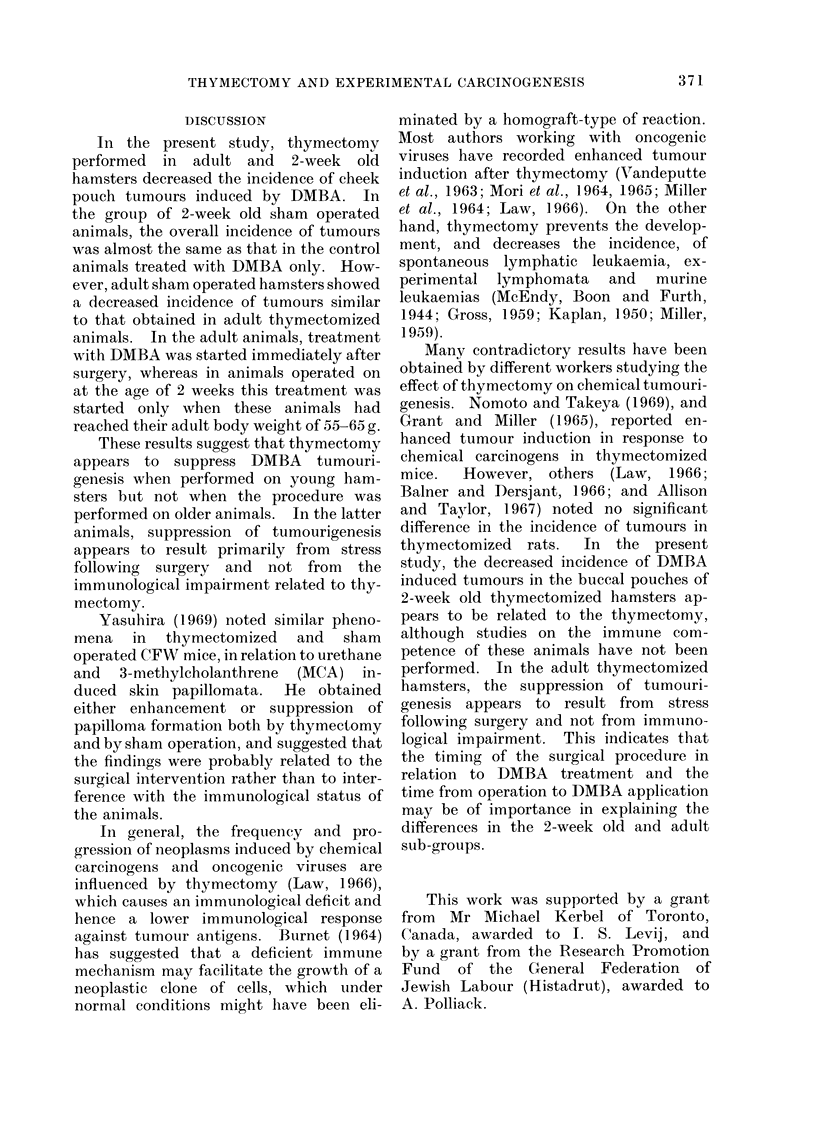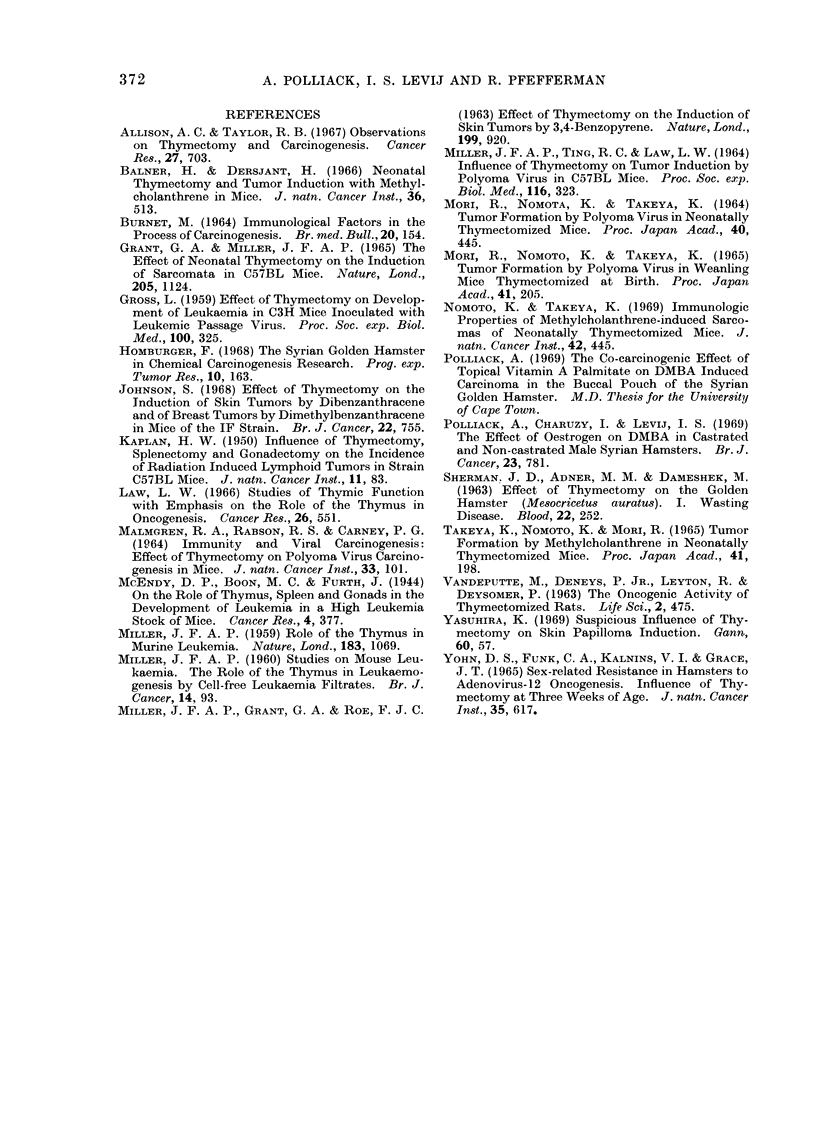# Observations on the Effect of Thymectomy or Chemical Carcinogenesis in the Hamster Cheek Pouch

**DOI:** 10.1038/bjc.1972.48

**Published:** 1972-10

**Authors:** A. Polliack, I. S. Levij, R. Pfefferman

## Abstract

The effects of thymectomy and sham operation on 9,10-dimethyl-1,2-benzanthracene (DMBA) induced tumours of the hamster cheek pouch were studied in Syrian golden hamsters. The incidence of carcinomata and papillomata with intra-epithelial carcinoma (atypical papillomata) in these animals was compared with that in control animals treated with DMBA alone, without surgical intervention.

In 32 non-operated control animals, the average tumour yield after 12 weeks' DMBA application was 2·13 carcinomata and 1·22 atypical papillomata per treated pouch. In 14 animals thymectomized at the age of 2 weeks, the tumour yield was 0·21 and 0·36, respectively, and in 10 animals thymectomized when adult, it was 0 and 0·1 respectively. In 9 animals sham operated on at the age of 2 weeks, an average of 1·56 carcinomata and 1·22 atypical papillomata were found, but in 13 animals which were sham operated when adult, the tumour yield was 0·54 and 0·15 per treated pouch, respectively.

The results suggest that the time of thymectomy in relation to DMBA treatment may be of importance and that thymectomy, when performed in 2-week old hamsters, inhibits DMBA tumourigenesis. The major effect of thymectomy performed in adult hamsters appears to be related to stress following surgery.


*Br. J. Cancer* (1972) **26**, 368

### OBSERVATIONS ON THE EFFECT OF THYMECTOMY ON CHEMICAL CARCINOGENESIS IN THE HAMSTER CHEEK POUCH

A. POLLIACK,* I. S. LEVIJ† AND R. PFEFFERMAN‡

*From the Departments of Haematology,* Pathology† and Surgery B‡, Hadassah University Hospital and Hebrew University-Hadassah Medical School, Jerusalem, Israel*

Received 17 April 1972. Accepted, 23 June 1972

**Summary.—The effects of thymectomy and sham operation on 9,10-dimethyl-1,2-benzanthracene (DMBA) induced tumours of the hamster cheek pouch were studied in Syrian golden hamsters. The incidence of carcinomata and papillomata with intra-epithelial carcinoma (atypical papillomata) in these animals was compared with that in control animals treated with DMBA alone, without surgical intervention.**

**In 32 non-operated control animals, the average tumour yield after 12 weeks' DMBA application was 2·13 carcinomata and 1·22 atypical papillomata per treated pouch. In 14 animals thymectomized at the age of 2 weeks, the tumour yield was 0·21 and 0·36, respectively, and in 10 animals thymectomized when adult, it was 0 and 0·1 respectively. In 9 animals sham operated on at the age of 2 weeks, an average of 1·56 carcinomata and 1·22 atypical papillomata were found, but in 13 animals which were sham operated when adult, the tumour yield was 0·54 and 0·15 per treated pouch, respectively.**

**The results suggest that the time of thymectomy in relation to DMBA treatment may be of importance and that thymectomy, when performed in 2-week old hamsters, inhibits DMBA tumourigenesis. The major effect of thymectomy performed in adult hamsters appears to be related to stress following surgery.**

TUMOUR induction with oncogenic viruses is enhanced in neonatally thymectomized animals (Vandeputte *et al.*, 1963; Mori, Nomoto and Takeya, 1964; Nomoto *et al.*, 1965; Miller, Ting and Law, 1964; Malmgren, Rabson and Carney, 1964; Yohn *et al.*, 1965). However, contradictory results have been obtained concerning the effect of thymectomy on chemical carcinogenesis, since some authors have recorded enhanced tumour induction (Miller, Grant and Roe, 1963; Grant and Miller, 1965; Nomoto and Takeya, 1969), whereas others (Law, 1966; Balner and Dersjant, 1966; Allison and Taylor, 1967) have not noted any significant difference in the incidence of tumours after neonatal thymectomy. In addition, only a few reports are available concerning thymectomy and chemically induced epithelial tumours (Miller *et al.*, 1963; Allison and Taylor, 1967; Johnson, 1968; Yasuhira, 1969).

In the light of these conflicting results, and the paucity of data concerning thymectomy and epithelial tumourigenesis, an experiment was designed to study the effect of neonatal and adult thymectomy on the development of hamster cheek pouch carcinomata. Until now, no such studies have been performed on this experimental model (Homburger, 1968; Polliack, 1969).

### MATERIALS AND METHODS

*Animals.*—Male Syrian golden hamsters of a local strain were used. The animals were housed in metal cages and after the surgical procedures had been performed were kept in pairs and fed Purina laboratory chow pellets and drinking water *ad libitum*. Thymectomy was performed either at the age of 2 weeks or in adult hamsters at the age of $1\frac{1}{2}$–2 months, their body weight ranging from 55 to 65 g.

*Thymectomy.*—Animals were thymectomized under ether anaesthesia using an adaptation of Miller's technique (Miller, 1960). Control hamsters were sham thymectomized by performing the identical surgical procedures except for actual removal of the thymus. Proof of successful thymectomy was confirmed at the end of the experiment, at autopsy. The absence of all thymic mediastinal tissue was determined by gross inspection and where doubtful by microscopic sectioning.

*Carcinogen.*—9, 10-dimethyl-1, 2-benzanthracene (DMBA) was dissolved in liquid paraffin (sp. gr. 1·0). Solutions of 0·5% w/v were prepared once a month and kept in dark bottles at room temperature. The solution was applied to the right cheek pouches of the experimental animals 3 times per week for 3 months, using a fine brush. The brush was dipped into the liquid, excess was allowed to drip off, and the pouch was then stroked firmly several times along its entire length. A preliminary test had shown that in this manner approximately 0·2 ml of the liquid, containing 1 mg of DMBA, was deposited in the pouch at each painting.

*Experiment.*—Sixty animals were initially operated upon. Fourteen of these did not survive the immediate post-operative period; 7 neonates developed either wasting disease similar to that described by Sherman, Adner and Dameshek (1963) or intercurrent infection and died, and 7 adult hamsters also died post-operatively from similar causes.

After operation the 60 animals were divided into the following 4 groups of 15 animals, and these numbers were subsequently reduced as shown by the deaths from causes described above.

Group 1. 15 animals thymectomized at the age of 6–8 weeks (10 survivors).
Group 2. 15 animals thymectomized at the age of 2 weeks (14 survivors).
Group 3. 15 animals sham-thymectomized at the age of 6–8 weeks (13 survivors).
Group 4. 15 animals sham-thymectomized at the age of 2 weeks (9 survivors).

A further 40 animals received topical treatment with DMBA alone, (Group 5, 32 survivors) and underwent no surgical procedure at all.

Groups 1 and 3 were treated with the carcinogen immediately after surgery was performed. Groups 2 and 4 were treated with the carcinogen when they reached the weight of Groups 1 and 3, *i.e.* 55–65 g.

*Autopsies.*—After the above treatments, the animals were killed, autopsied and the tumours present in the cheek pouches were counted and measured. These tumours, non-tumourous areas of all cheek pouches, the regional lymph nodes and the internal organs were examined histologically.

*Histological criteria.*—The most frequently encountered cheek pouch lesions will be briefly defined as in previous studies (Polliack, Charuzy and Levij, 1969).

1. Invasive squamous cell carcinoma: tumour showing marked cellular and nuclear pleomorphism, loss of normal epithelial polarity and increased number of mitoses, and disappearance of the basal membrane with invasion of the lamina propria by tumour cells.

2. Intra-epithelial carcinoma: lesion characterized by cellular and nuclear changes as above, but with an intact basal membrane and without invasive growth. Areas of intra-epithelial carcinoma were often found in otherwise benign squamous cell papillomata, but they were also present in macroscopically non-tumourous cheek pouch mucosa.

3. Atypical papilloma: papillomatous tumour with areas of intra-epithelial carcinoma as described above.

The number of squamous cell carcinomata and papillomata in each pouch could be determined exactly, but it was impossible to record accurately the number of intra-epithelial carcinomata since these lesions did not present as tumours macroscopically and thus were detected only during the histological examination. Their frequency was estimated during examination of many sections, and the impression gained during this study was expressed as + + when many foci were present in each animal, and as + when only a small number of these lesions was found.

The internal organs and regional lymph nodes showed no histological or macroscopical changes in any of the animals.

TABLE I.—*Incidence of Cheek Pouch Tumours after Topical Administration of DMBA for 12 Weeks in Thymectomized, Sham Operated and Non-thymectomized Hamsters*

| Groups | No. of animals | No. with carcinoma | Total no. of carcinomata | Average no. carcinomata animals | No. with atypical papillomata | Total no. of atypical papillomata | Average No. of atypical papillomata animals | Intra-epithelial carcinoma |
|---|---|---|---|---|---|---|---|---|
| 1<br>Thymectomized adult hamsters | 10 | 0 | 0 | 0 | 1 | 1 | 0·10 | + |
| 2<br>Thymectomized 2-week old hamsters | 14 | 3 | 3 | 0·21 | 4 | 5 | 0·36 | + |
| 3<br>Sham operated adult hamsters | 13 | 6 | 7 | 0·54 | 2 | 2 | 0·15 | + |
| 4<br>Sham operated 2-week old hamsters | 9 | 6 | 14 | 1·56 | 4 | 11 | 1·22 | + |
| 5<br>Non-thymectomized hamsters | 32 | 26 | 68 | 2·13 | 24 | 39 | 1·22 | ++ |

FIG. 1.—Tumour incidence in thymectomized, sham operated and control hamsters after 12 weeks' treatment with DMBA.

### RESULTS

The results are summarized in Table I and the incidence of carcinomata and atypical papillomata in the different groups is compared in Fig. 1.

The tumour incidence in adult thymectomized animals was much lower than in non-operated controls, but sham operation in adult animals also appeared to suppress tumour formation. In animals operated on at the age of 2 weeks, thymectomy caused marked suppression of tumourigenesis, but sham operation performed on animals of this age had no evident effect.

### DISCUSSION

In the present study, thymectomy performed in adult and 2-week old hamsters decreased the incidence of cheek pouch tumours induced by DMBA. In the group of 2-week old sham operated animals, the overall incidence of tumours was almost the same as that in the control animals treated with DMBA only. However, adult sham operated hamsters showed a decreased incidence of tumours similar to that obtained in adult thymectomized animals. In the adult animals, treatment with DMBA was started immediately after surgery, whereas in animals operated on at the age of 2 weeks this treatment was started only when these animals had reached their adult body weight of 55–65 g.

These results suggest that thymectomy appears to suppress DMBA tumourigenesis when performed on young hamsters but not when the procedure was performed on older animals. In the latter animals, suppression of tumourigenesis appears to result primarily from stress following surgery and not from the immunological impairment related to thymectomy.

Yasuhira (1969) noted similar phenomena in thymectomized and sham operated CFW mice, in relation to urethane and 3-methylcholanthrene (MCA) induced skin papillomata. He obtained either enhancement or suppression of papilloma formation both by thymectomy and by sham operation, and suggested that the findings were probably related to the surgical intervention rather than to interference with the immunological status of the animals.

In general, the frequency and progression of neoplasms induced by chemical carcinogens and oncogenic viruses are influenced by thymectomy (Law, 1966), which causes an immunological deficit and hence a lower immunological response against tumour antigens. Burnet (1964) has suggested that a deficient immune mechanism may facilitate the growth of a neoplastic clone of cells, which under normal conditions might have been eliminated by a homograft-type of reaction. Most authors working with oncogenic viruses have recorded enhanced tumour induction after thymectomy (Vandeputte *et al.*, 1963; Mori *et al.*, 1964, 1965; Miller *et al.*, 1964; Law, 1966). On the other hand, thymectomy prevents the development, and decreases the incidence, of spontaneous lymphatic leukaemia, experimental lymphomata and murine leukaemias (McEndy, Boon and Furth, 1944; Gross, 1959; Kaplan, 1950; Miller, 1959).

Many contradictory results have been obtained by different workers studying the effect of thymectomy on chemical tumourigenesis. Nomoto and Takeya (1969), and Grant and Miller (1965), reported enhanced tumour induction in response to chemical carcinogens in thymectomized mice. However, others (Law, 1966; Balner and Dersjant, 1966; and Allison and Taylor, 1967) noted no significant difference in the incidence of tumours in thymectomized rats. In the present study, the decreased incidence of DMBA induced tumours in the buccal pouches of 2-week old thymectomized hamsters appears to be related to the thymectomy, although studies on the immune competence of these animals have not been performed. In the adult thymectomized hamsters, the suppression of tumourigenesis appears to result from stress following surgery and not from immunological impairment. This indicates that the timing of the surgical procedure in relation to DMBA treatment and the time from operation to DMBA application may be of importance in explaining the differences in the 2-week old and adult sub-groups.

This work was supported by a grant from Mr Michael Kerbel of Toronto, Canada, awarded to I. S. Levij, and by a grant from the Research Promotion Fund of the General Federation of Jewish Labour (Histadrut), awarded to A. Polliack.

### REFERENCES

ALLISON, A. C. & TAYLOR, R. B. (1967) Observations on Thymectomy and Carcinogenesis. *Cancer Res.*, **27**, 703.

BALNER, H. & DERSJANT, H. (1966) Neonatal Thymectomy and Tumor Induction with Methylcholanthrene in Mice. *J. natn. Cancer Inst.*, **36**, 513.

BURNET, M. (1964) Immunological Factors in the Process of Carcinogenesis. *Br. med. Bull.*, **20**, 154.

GRANT, G. A. & MILLER, J. F. A. P. (1965) The Effect of Neonatal Thymectomy on the Induction of Sarcomata in C57BL Mice. *Nature, Lond.*, **205**, 1124.

GROSS, L. (1959) Effect of Thymectomy on Development of Leukaemia in C3H Mice Inoculated with Leukemic Passage Virus. *Proc. Soc. exp. Biol. Med.*, **100**, 325.

HOMBURGER, F. (1968) The Syrian Golden Hamster in Chemical Carcinogenesis Research. *Prog. exp. Tumor Res.*, **10**, 163.

JOHNSON, S. (1968) Effect of Thymectomy on the Induction of Skin Tumors by Dibenzanthracene and of Breast Tumors by Dimethylbenzanthracene in Mice of the IF Strain. *Br. J. Cancer*, **22**, 755.

KAPLAN, H. W. (1950) Influence of Thymectomy, Splenectomy and Gonadectomy on the Incidence of Radiation Induced Lymphoid Tumors in Strain C57BL Mice. *J. natn. Cancer Inst.*, **11**, 83.

LAW, L. W. (1966) Studies of Thymic Function with Emphasis on the Role of the Thymus in Oncogenesis. *Cancer Res.*, **26**, 551.

MALMGREN, R. A., RABSON, R. S. & CARNEY, P. G. (1964) Immunity and Viral Carcinogenesis: Effect of Thymectomy on Polyoma Virus Carcinogenesis in Mice. *J. natn. Cancer Inst.*, **33**, 101.

McENDY, D. P., BOON, M. C. & FURTH, J. (1944) On the Role of Thymus, Spleen and Gonads in the Development of Leukemia in a High Leukemia Stock of Mice. *Cancer Res.*, **4**, 377.

MILLER, J. F. A. P. (1959) Role of the Thymus in Murine Leukemia. *Nature, Lond.*, **183**, 1069.

MILLER, J. F. A. P. (1960) Studies on Mouse Leukaemia. The Role of the Thymus in Leukaemogenesis by Cell-free Leukaemia Filtrates. *Br. J. Cancer*, **14**, 93.

MILLER, J. F. A. P., GRANT, G. A. & ROE, F. J. C. (1963) Effect of Thymectomy on the Induction of Skin Tumors by 3,4-Benzopyrene. *Nature, Lond.*, **199**, 920.

MILLER, J. F. A. P., TING, R. C. & LAW, L. W. (1964) Influence of Thymectomy on Tumor Induction by Polyoma Virus in C57BL Mice. *Proc. Soc. exp. Biol. Med.*, **116**, 323.

MORI, R., NOMOTA, K. & TAKEYA, K. (1964) Tumor Formation by Polyoma Virus in Neonatally Thymectomized Mice. *Proc. Japan Acad.*, **40**, 445.

MORI, R., NOMOTO, K. & TAKEYA, K. (1965) Tumor Formation by Polyoma Virus in Weanling Mice Thymectomized at Birth. *Proc. Japan Acad.*, **41**, 205.

NOMOTO, K. & TAKEYA, K. (1969) Immunologic Properties of Methylcholanthrene-induced Sarcomas of Neonatally Thymectomized Mice. *J. natn. Cancer Inst.*, **42**, 445.

POLLIACK, A. (1969) The Co-carcinogenic Effect of Topical Vitamin A Palmitate on DMBA Induced Carcinoma in the Buccal Pouch of the Syrian Golden Hamster. *M.D. Thesis for the University of Cape Town.*

POLLIACK, A., CHARUZY, I. & LEVIJ, I. S. (1969) The Effect of Oestrogen on DMBA in Castrated and Non-castrated Male Syrian Hamsters. *Br. J. Cancer*, **23**, 781.

SHERMAN, J. D., ADNER, M. M. & DAMESHEK, M. (1963) Effect of Thymectomy on the Golden Hamster (*Mesocricetus auratus*). I. Wasting Disease. *Blood*, **22**, 252.

TAKEYA, K., NOMOTO, K. & MORI, R. (1965) Tumor Formation by Methylcholanthrene in Neonatally Thymectomized Mice. *Proc. Japan Acad.*, **41**, 198.

VANDEPUTTE, M., DENEYS, P. JR., LEYTON, R. & DEYSOMER, P. (1963) The Oncogenic Activity of Thymectomized Rats. *Life Sci.*, **2**, 475.

YASUHIRA, K. (1969) Suspicious Influence of Thymectomy on Skin Papilloma Induction. *Gann*, **60**, 57.

YOHN, D. S., FUNK, C. A., KALNINS, V. I. & GRACE, J. T. (1965) Sex-related Resistance in Hamsters to Adenovirus-12 Oncogenesis. Influence of Thymectomy at Three Weeks of Age. *J. natn. Cancer Inst.*, **35**, 617.